# Cancer-secreted exosomal miR-1246 promotes colorectal cancer liver metastasis by activating hepatic stellate cells

**DOI:** 10.1186/s10020-025-01112-w

**Published:** 2025-02-20

**Authors:** Xiaolong Liu, Jialong Liu, Xuanyin Wang, Yang Zou, Xinyi Tao, Jingyu Li, Mengnan Ye, Wanbei Xu, Yunyao Deng, Lixin Liu, Jingbo Sun, Qingling Zhang

**Affiliations:** 1 Department of General Surgery, The Third Affiliated Hospital of Southern Medical University, 183 West Zhongshan Avenue, Guangzhou, Guangdong 510630 People’s Republic of China; 2 Department of Pathology, Guangdong Provincial People’s Hospital (Guangdong Academy of Medical Sciences), Southern Medical University, No.106, Zhongshan 2 Road, Guangzhou, Guangdong 510080 People’s Republic of China

**Keywords:** CRLM, Exosome, miR-1246, HSCs, Cholesterol metabolism

## Abstract

Colorectal cancer liver metastases (CRLM) are the primary cause of mortality in colorectal cancer (CRC) patients, highlighting the importance of understanding the underlying mechanisms. The tumor microenvironment (TME) and its interaction with tumor cells play a crucial role in CRLM progression. Notably, the stability and peak levels of tumor-derived exosomal miRNAs facilitate intercellular communication in the TME. Hepatic stellate cells (HSCs), key liver mesenchymal cells, constitute about 33% of the liver's nonsolid cell population and exhibit plasticity. However, the specific role of tumor-derived exosomal miRNAs in the crosstalk between HSCs and tumor cells during the CRLM process remains unclear. We studied CRC-secreted exosomal miR-1246 and its impact on HSCs, as well as its effects on CRC cell proliferation and metastasis. Our findings demonstrate that CRC-secreted exosomal miR-1246 can be internalized by HSCs, leading to their activation and facilitating the metastatic potential of CRC cells. Mechanistically, exosomal miR-1246 targets INSIG1, resulting in SREBP2 nucleation and cholesterol metabolism alterations. This accumulation of free cholesterol (FC) regulates the TLR4/NF-κB/TGF-β pathway, promoting HSC activation. Activated HSCs, in turn, enhance liver metastasis of CRC cells through the TNFSF13/TNFRSF13B axis. Our study reveals the role of CRC-secreted exosomal miR-1246 in triggering HSC activation and reprogramming the TME, ultimately facilitating liver metastasis in CRC patients. Exosomal miR-1246 could serve as a potential non-invasive biomarker for predicting colorectal cancer liver metastasis, enhancing our understanding of CRC-associated liver metastases.

**Graphical abstract:**

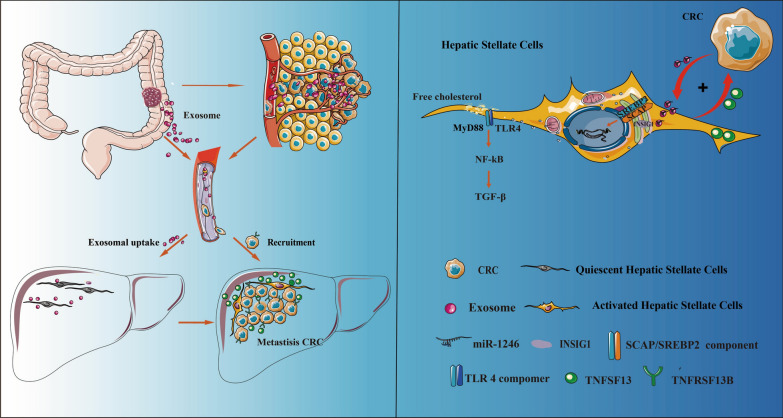

**Supplementary Information:**

The online version contains supplementary material available at 10.1186/s10020-025-01112-w.

## Introduction

Colorectal cancer (CRC) has ascended to the position of the second most lethal cancer worldwide (Sedlak et al. 2023). Early intervention in CRC can yield beneficial outcomes, however, a substantial 25–50% of CRC patients are vulnerable to further metastatic complications (Wang et al. 2023). Approximately 15–25% of CRC patients are estimated to develop synchronous liver metastases at their first diagnosis, and 18–25% of CRC patients eventually develop synchronous liver metastases within 5 years of initial diagnosis (Wang et al. 2021).

Current treatment strategies for colorectal cancer liver metastases (CRLM) integrate systemic therapies with surgical and interventional approaches (Czauderna et al. 2021). Despite advancements in surgical techniques and systemic therapies, only 10–20% of patients are eligible for curative resection, with a five-year survival rate as low as 30% (Takahashi and Berber 2020). This shortfall is primarily due to limited understanding of the mechanisms underlying CRLM.

Emerging evidence on CRLM demonstrates that CRC can remodel distant organs by releasing exosomes, which help create a favorable tumor microenvironment (TME) for tumor colonization (Guo et al. 2019). Exosomes, acting as kay communication facilitators, are implicated in the malignant progression of colorectal cancers. Concurrently, several studies have highlighted that the activated hepatic stellate cells (aHSCs) are a common biological feature in both primary and metastatic liver tumors (Filliol et al. 2022; Costa-Silva et al. 2015).

HSCs exhibit remarkable plasticity, enabling them to transition from a quiescent to an activated state (Tan et al. 2020). Notably, aHSCs can promote a fibrotic liver microenvironment, which in turn supports the growth and invasion of Pancreatic Ductal Adenocarcinoma (PDAC) or CRC cells (Xie et al. 2022; Tsuchida and Friedman 2017). Therefore, exploring the interactions between CRC cells and HSCs, and understanding their underlying mechanisms, could be crucial for the prevention or treatment of CRLM.

Exosomes, a subtype of extracellular vesicles (EVs), are small vesicles range from 30 to 100 nm in diameter, generated by eukaryotic cells. Enclosed in a lipid bilayer, they carry a variety of biological molecules and act as essential mediators for intercellular communication b–y transporting proteins, lipids, DNA, RNA, and miRNAs (Kalluri and Lebleu 2020; Lai et al. 2022). Once this molecular cargo is taken up by a recipient cell, it can alter the recipient cell’s phenotype (Wortzel et al. 2019), making exosomes pivotal players in intercellular communication. Tumor cells exploit this communication system to remodel recipient cells, initiating oncogenic processes such as angiogenesis, immunomodulation, metastasis, and proliferation (Hoshino et al. 2015; Zhang and Yu 2019). The metastatic microenvironment formed through exosome-mediated interactions, known as the pre-metastatic niche, facilitates the colonization and malignant progression of circulating tumor cells. In the liver, hepatic stellate cells (HSCs) are abundant, and their activity is directly linked to the development of CRLM (Zhao et al. 2022). The remarkable plasticity of HSCs suggests they may be regulated by exosomes derived from CRC cells. However, the specific pathways through which miRNAs contribute to HSCs activation and promote CRLM remain unclear.

Studies have shown that high expression of miR-1246 in various digestive tumors, including CRC and pancreatic cancer, promotes tumor progression and resistance to chemotherapy. SG.et.al. proposed that miR-1246 is part of a p53-associated intercellular network that enhances chemoresistance by increasing pancreatic tumor stemness through the regulation of CCNG2 (Cooks et al. 2018). Furthermore, methylation by METTL3 modifies pri-miR-1246, promoting its maturation and regulating SPRED2, which drives colorectal tumors metastasis (Peng et al. 2019).

In our experiment, we observed notably high expression of miR-1246 in both the primary lesion and serum exosomes of CRC patients, which was positively correlated with the incidence of CRLM. Additionally, we found a significant increase in the number of aHSCs in the CRLM mesenchyme. As a result, we hypothesize that CRC-secreted exosomal miR-1246 travels to distant liver tissue and may mediate CRC liver metastasis through the activation of HSCs.

In this study, we found that CRC-secreted exosomal miR-1246 suppresses INSIG1 expression and promotes SREBP2 nucleation, thereby regulating cholesterol metabolism. The resulting accumulation of free cholesterol activates the TLR4/NF-κB/TGF-β pathway, which in turn leads to the activation of HSCs. These activated HSCs further enhance the malignant progression of CRC through the TNFSF13/TNFRSF13B axis. The interaction between CRC cells and HSCs reprograms cholesterol metabolism within the Tumor Microenvironment, driving the progression of colorectal cancer liver metastases (CRLM).

The interaction between CRC and HSCs reprograms cholesterol metabolism within the tumor microenvironment, driving the progression of colorectal cancer liver metastases. Our findings highlight a novel biochemical index that could serve as both a predictive marker and a therapeutic target, offering potential strategies for addressing CRLM.

## Material and methods

### Microarray analysis and human tissue samples

miRNA expression in normal and CRC tissues with or without metastases was analyzed using the Human miRNA Paraflo™ Array (LC Sciences, CA, USA). The experiments involved research protocols and informed consent were approved by the Ethics Committee of the Third Affiliated Hospital of Southern Medical University. (Guangzhou, China). Informed consent was signed by all patients. All clinical samples were obtained from the Third Affiliated Hospital of Southern Medical University.

### Cell lines

Human hepatic stellate cell (LX2), human embryonic kidney 293T cell line (HEK293T), human colon cell line (FHC), human CRC cell lines (HT29, HCT116, SW620, SW480, DLD1, RKO, and LOVO) were purchased from the Chinese Academy of Medical Sciences Cell Bank (Shanghai, China). Cells were cultured in Duchenne modified medium (DMEM; Gibco, Carlsbad, CA, United States) containing 10% fetal bovine serum (FBS; Thermo Scientific, Waltham, MA, United States), 100 IU/mL penicillin G, and 100 µg/mL streptomycin (Invitrogen Life Technologies, Carlsbad, CA, United States).

### RNA interference and plasmids

The mimic for miR-1246 overexpression, the overexpression plasmid, and the lentivirus for gene knockdown were synthesized by Genechem (Guangzhou, Guangdong, China). The human pSV40-INSIG1 overexpression plasmid was also provided by Genechem. Detailed sequences of these plasmids and viruses are available in Supplementary Table 1.

### Exosome isolation, characterization, and treatment

CRC cells were cultured in DMEM medium supplemented with 1–2% de-exosomalized fetal bovine serum. De-exosomalized fetal bovine serum is purified by overnight centrifugation with a 110,000*g* superspeed centrifuge. 48 h later the conditioned medium is centrifuged at 500*g* and 16,800*g* at 4 °C and the supernatant is collected. The supernatant was passed through a 0.22 um filter (Millipore) and then centrifuged at 110,000*g* at 4 °C for one hour. The exosomes obtained were assayed with the BCA Protein Assay Kit (KeyGEN BioTECH). Exosomes were fixed with 2% paraformaldehyde, then stained and observed on a transmission electron microscope (Hitachi H-7500).

### Exosome labeling

Fluorescent labeling was performed with PKH67 membrane dye (Sigma). Then 2–5 µg of ultracentrifugated and resuspended exosomes were incubated with 2 × 10^5^ recipient cells for 48–72 h.

### In situ hybridization and fluorescence in situ hybridization

Paraffin-embedded tissue blocks were cut into 2.5 μm sections. Tissue sections were subjected to fluorescence in situ hybridization or situ hybridization (ISH, FISH) using an ISH kit (Bosterbio, USA) for miR-1246-cy3 or miR-1246 horseradish peroxidase-modified detection probe (Biolink, Guangzhou, USA). Fluorescence intensities were analyzed with ImageJ.

### Immunohistochemistry

For immunohistochemistry (IHC), paraffin-embedded tissue blocks were cut into 2.5 μm sections. Routine dewaxing, fixation, immersion in 3% hydroxide solution, and antigen repair followed by goat serum closure were performed and then slides were incubated with primary antibodies α-SMA (abcam ab7817, 1:50,000 dilution), Collagen Type I (proteintech, 14695-1-AP, 1:200 dilution), TNFRSF13B (Affinity, DF14079, 1:200 dilution) and KI67 (abcam, ab15580, 1:1000 dilution) at 4 °C and corresponding horseradish peroxidase-labeled secondary antibody, developed with DAB and counterstained with hematoxylin.

### Immunofluorescence

Tissue sections or cells were fixed in 4% paraformaldehyde, blocked and incubated with primary antibodies against α-SMA (abcam ab7817, 1:800 dilution), Collagen Type I (proteintech, 14695-1-AP, 1:200 dilution), INSIG1 (Abclon, A23531, 1:200 dilution), SREBP2 (SANTA, sc-13552, 1:100 dilution), CK20 (proteintech, 17329-1-AP, 1:200 dilution), Phalloidin-iFluor 594 (abcam, ab176757, 1:1000 dilution) at 4 °C overnight and corresponding fluorescent secondary antibodies. Photographs were taken under an LSM900 laser confocal microscope. Fluorescence intensities were analyzed with ImageJ.

### RNA isolation, reverse transcription, and quantitative real-time PCR

Total RNA was extracted using Trizol (Invitrogen, U.S.A.) Polyadenylation and reverse transcription (RT) of total RNA were performed using a ThermoScriptTM RT-PCR system (Invitrogen), Mir-X™ miRNA First-Strand Synthesis Kit for miRNAs (Clontech Laboratories, USA) or PrimeScript™ RT Master Mix for general genes (Clontech Laboratories, USA). Real-time polymerase chain reaction (PCR) analysis was performed on the BIO-RED system with SYBR Green PCR master mix (Applied Biosystems, United States). The sequences of all indicated primers are listed in Supplementary Table 2.

### Western blotting

All samples were lysed in RIPA lysis buffer, quantified by the BCA Protein Detection Kit (Pierce, KeyGEN BioTECH, China), separated on SDS-PAGE gels, transferred to PVDF membranes, intubated in 5% skim milk powder, rabbit antibodies CD54 and CD63 (1:1000, CST), INSIG1, GAPDH, (1:1000, Abcam) and murine antibodies ColI, TGF-Β, NFκb p65, Phospho-NFκb p65 (1:1000, Proteintech), SCAP, and SREBP2, TLR4, PU.1, SP1 (1:1000, SANTA) at 4 °C overnight. And incubation with HRP-conjugated secondary antibody (anti-rabbit IgG/anti-mouse IgG, 1:15,000, CST.) was followed. It was imaged with an enhanced chemiluminescence detection system (GV 6000pro, China).

### Migration assay and EDU assay

For the migration assay, LX2 cells pre-treated with exosomes were seeded into the lower chamber of Transwell (Corning, China) and medium containing 2% FBS was added. Colorectal cancer cell lines were seeded in the upper chamber of Transwell. Staining and counting were performed after 3–4 days of co-culture. For EDU experiments, a thymine nucleoside analog, is incorporated into DNA during cell proliferation. Cell proliferation is detected by forming EdU-DNA complexes and labeling them with a fluorescent dye. CRC cells were co-cultured with exosome-pretreated LX2 and then photographed under the Leica fluorescence microscope after 2–3 days by using BeyoClick™ EDU-594 Kit.

### Luciferase activity assay

The INSIG1 3′ UTR plasmid: 3utr (INSIG1-3UTR (mir1246)) and 3utr (INSIG1-3UTR (mir1246)-mut) (Genechem Company, Guangzhou, China) was co-transfected with miR-1246 mimic into cells using Lipofectamine 2000 (Invitrogen). The cells were analyzed by Dual-Luciferase Luciferase activity was measured 48 h after transfection using the Dual-Luciferase reporter assay system (Promega). All experiments were performed in triplicate and each experiment was repeated three times.

### Animal models

Five-week-old female thymus-free BALB/c-nu/nu mice were purchased from the Guangdong Animal Experiment Center (Guangzhou, China). CRC cells were implanted in the spleen to create a metastasis model. The mice then received 5 µg of exosomes bi-weekly intraperitoneal injections of exosomes for 7 weeks before mice were sacrificed for sample collection and evaluation. All procedures were approved by the Institutional Animal Care and Use Committee.

### Statistical analysis

Statistical analysis was performed using Prism 8.0 (GraphPad Software, San Diego, CA, United States). Student's t-test, One-Way and Two-Way ANNOVA were used to assess the significance of differences between groups. All data are expressed as mean ± standard deviation (SD). P < 0.05 was considered to be statistically significant. Survival curves were generated using R's 'survival' package, while Pearson correlation analysis was used for correlation between groups. GO (Gene Ontology) analysis of biological process (BP) was performed on mRNA sequencing data. Adobe Illustrator CC, Adobe, Photoshop CC, and Image J software were used for figure presentation.

## Results

### Up-regulation of exosomal miR-1246 associated with liver metastatic progression and HSCs activation in CRLM patients

**Fig. 1:**
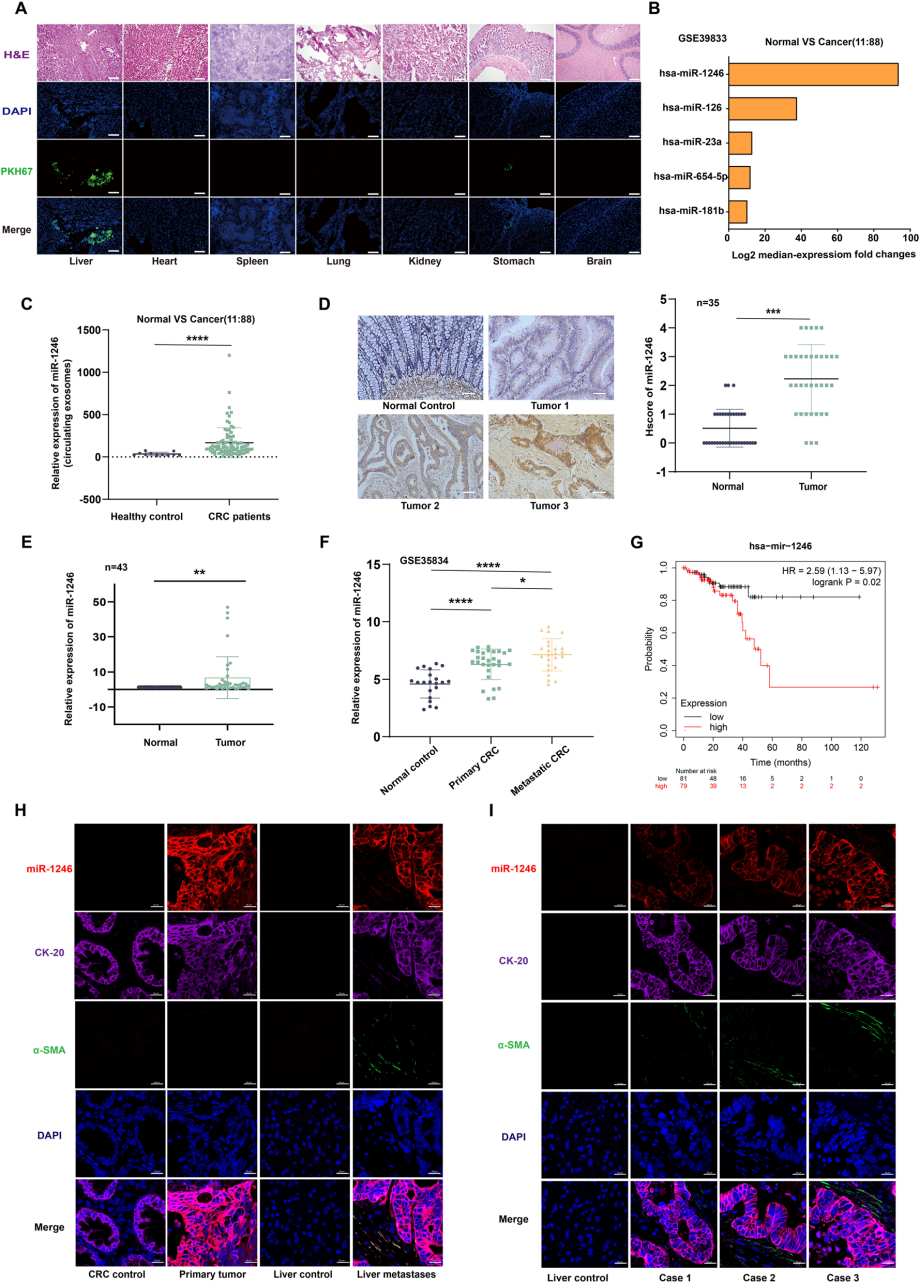
Up-regulation of exosomal miR-1246 associated with liver metastatic progression and HSCs activation in CRLM patients. **A** Intraperitoneal injection of CRC-derived PKH67-labeled exosomes recruited in the liver, stomach, and lung of mice. Scale bars: 100 µm. **B** Among the differentially expressed miRNAs, the top five (miR-1246, miR-126, miR-23a, miR-654-5p, and miR-181b) are displayed. (accession number: GSE39833). **C** The scatter plots demonstrate the relative expression levels of miR-1246 in CRC serum exosomes versus healthy individuals. **D** ISH highlights miR-1246 in 35 fresh CRC tissue sections. Scale bars: 100 µm. Mean ± SEM; n = 35. **E** RT-PCR analysis of miR-1246 expression in 43 paired CRC samples. Mean ± SEM; n = 3. **F** The miRNA array data of miR-1246 levels in healthy populations, primary CRC, and metastatic CRC are shown (accession number: GSE35834). **G** Overexpressed miR-1246 levels were significantly associated with Overall Survival in CRC patients (p < 0.05). **H**–**I** Representative immunofluorescence staining images and qualification of miR-1246 (labeled with cy3, red) in CRC liver metastatic cells (labeled with CK20, purple) and activated hepatic stellate cells (labeled with α-SMA, green) from CRC negative control, primary tumor, liver negative control and liver metastases. Scale bars: 20 µm

A multitude of studies have highlighted the significant role of CRC exosomes in tumor progression and metastasis (Zhao et al. 2020; Tian et al. 2021). To assess whether these exosomes can be taken up by the liver and remain functional, we labeled exosomes from HCT116 cells with PKH67 lipid dye and injected them into the peritoneal cavity of nude mice. Our in vivo tracer experiments revealed significant absorption of the intraperitoneally injected exosomes by the liver, stomach, and lungs (Fig. 1A). This finding indicates that CRC exosomes are highly targetable to the liver, which may explain the high incidence of CRLM.

Next, we analyzed serum exosomes from both CRC patients and healthy individuals using the GSE39833 dataset, aiming to identify miRNA-driven mechanisms that contribute to CRC progression and prognosis. Among the differentially expressed miRNAs, the top five (miR-1246, miR-126, miR-23a, miR-654-5p, and miR-181b) are shown (Fig. 1B). Notably, miR-1246 was prominently overexpressed in CRC serum exosomes (Fig. 1C).

In situ hybridization (ISH) performed on 35 paired paraffin-embedded CRC tissue samples further confirmed significant overexpression of miR-1246 in CRC tissues compared to normal colorectal mucosa (Fig. 1D). Additionally, our analysis of the TCGA pan-cancer miRNA matrix revealed that miR-1246 levels were markedly higher in CRC than in other tumor types (Supplementary Fig. 1A). Similarly, RT-PCR analysis of 43 paired fresh CRC tissue samples verified an increase in miR-1246 expression in CRC tissues (Fig. 1E). Interestingly, miR-1246 levels were closely associated with liver metastasis, as indicated by the GSE35834 dataset and ISH experiments (Fig. 1F, Supplementary Fig. 1B). Moreover, overexpression of miR-1246 was strongly correlated with poor prognosis in CRC patients (Fig. 1G). Notably, liver metastases from CRC frequently exhibited concurrent activation of hepatic stellate cells (HSCs) (Fig. 1H, Supplementary Fig. 1C). A positive correlation was observed between the expression of miR-1246 (red) in CRC liver metastatic cells (labeled with CK20, purple) and the activation of HSCs (labeled by α-SMA, green, Fig. 1I, Supplementary Fig. 1D). In summary, our analyses suggest a strong association between elevated miR-1246 expression in CRC, activation of HSCs, and the malignant progression of CRLM.

### CRC-secreted miR-1246 is transferred to active HSCs via exosomes

**Fig. 2:**
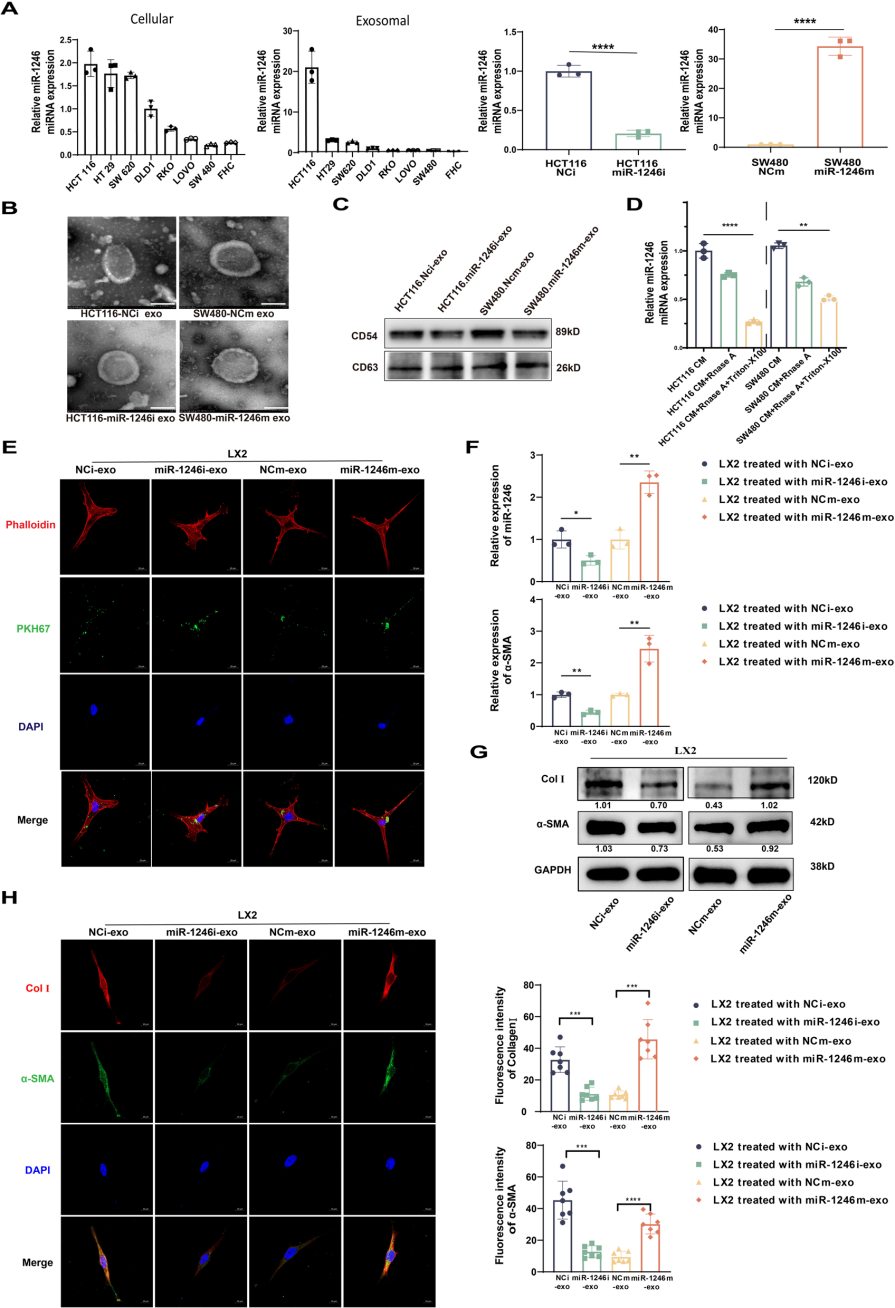
CRC-secreted miR-1246 is transferred to active HSCs via exosomes. **A** RT-PCR results display miR-1246 expression in CRC cell lines, exosomes, and modified SW480 and HCT116. Mean ± SEM; n = 3. **B** Morphological images of exosomes from HCT116 miR-1246i, SW480 miR-1246m, and controls via electron microscopy. Scale bars: 100 nm. **C** CD54 and CD63 exosome markers verified by Western blot. **D** miR-1246 in exosomes post alteration of membrane permeability and RNase A digestion confirmed by RT-PCR. Mean ± SEM; n = 3. **E** Confocal images of PKH67-labeled exosomes (green) after incubation with Phalloidin-labeled HSCs (red). Scale bars: 20 µm. **F** miR-1246 and α-SMA in HSCs treated with group-sourced exosomes analyzed by RT-PCR. Mean ± SEM; n = 3. **G** ColI and α-SMA activation validation via Western blot. **H** Confocal images and quantification of enhanced ColI (red) and α-SMA (green) in modified groups. Scale bars: 20 µm. Mean ± SEM

There is evidence supporting that miRNAs may be involved in intercellular communication via exosomes (Chen et al. 2021; Zhang et al. 2022a). Consequently, we speculated whether miR-1246 could traverse from CRC cells to HSCs via exosomes, potentially triggering the activation process of the latter. Using RT-PCR, we detected the endogenous expression of miR-1246 in CRC cell lines and corresponding exosomes, HCT116 miR-1246i was used to knock down miR-1246 in HCT116 cells with high expression levels, while SW480 miR-1246m was used for overexpression. (Fig. 2A). Transmission electron microscopy was employed to characterize exosomes derived from the conditioned media of HCT116-miR-1246i, SW480-miR-1246m, and controls, defining them as membrane-enveloped particles distinct in size, ranging from 50 to 100 nm (Fig. 2B). In addition, Western blot analysis confirmed the presence of exosome-positive markers CD54 and CD63 (Fig. 2C). Employing the notable property of Triton-X100 to modify the permeability of exosome membrane structures and RNA digestion by Rnase A, we determined the presence of miR-1246 in exosomes obtained from conditioned media. As anticipated, the addition of Triton-X100 further amplified the digestion of mir-1246 by Rnase A, supporting that miR-1246 primarily functions in intercellular communication through exosomes (Fig. 2D). In subsequent testing, PKH67-labeled exosomes (green) were incubated with Phalloidin-labeled Human hepatic stellate cell line (LX2, red). The uptake of PKH67-labeled exosomes by LX2 was clearly observable (Fig. 2E). A subsequent RT-PCR assay indicated that the expression of both miR-1246 and α-SMA in LX2 significantly increased following co-culture with SW480-miR-1246m exosomes, in comparison with the SW480-NCm exosomes group. This expression sharply decreased in the knockdown group (Fig. 2F). Moreover, Western blot and Immunofluorescence findings showed a surge in the expression of HSCs activation markers ColI and α-SMA upon treatment with SW480-miR-1246m exosomes while inhibiting the expression of miR-1246 had the inverse effect (Fig. 2G, H). In conclusion, our results suggest that CRC-secreted miR-1246 can transmit to HSCs via exosomes, provoking their transition from dormant to activated state.

### aHSCs promote CRC proliferation and migration

**Fig. 3:**
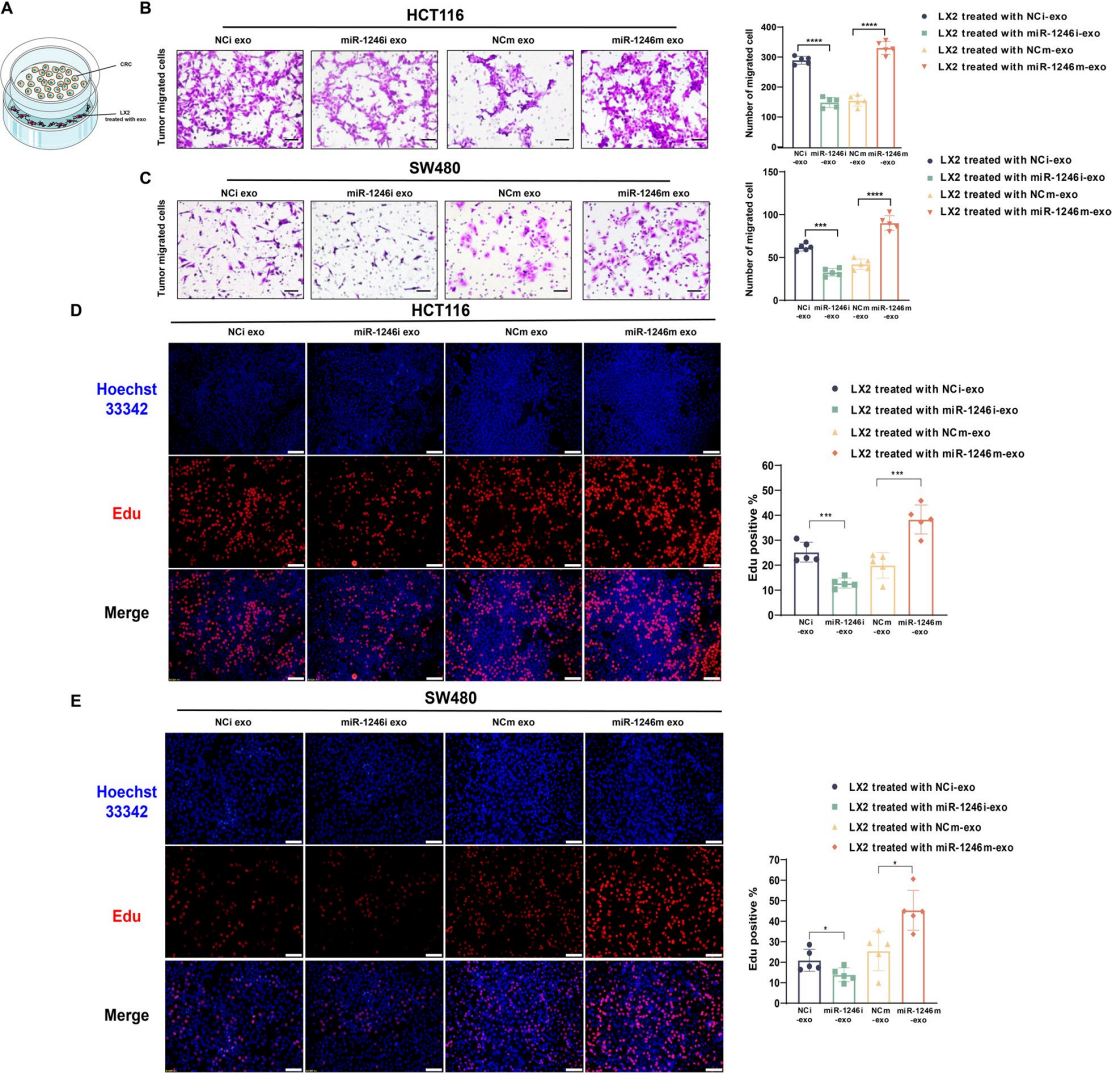
aHSCs promote CRC proliferation and migration. **A** Schematic of indirect co-culture system. **B**, **C** Migration enhancement of SW480 and HCT116 CRC cells after HSCs stimulated by CRC exosomes, assessed by transwell assay. Scale bars:50 µm, Mean ± SEM (n = 5). **D**, **E** Proliferation of CRC was quantified and visualized by EDU assay. Scale bars:100 µm, Mean ± SEM (n = 5)

Previous research intimates that the activation of HSCs is associated with liver metastasis and poor prognosis in CRC patients, with aHSCs contributing to the malignant progression of CRC by releasing a variety of cytokines and cytokine receptors (Zhao et al. 2022; Zhang et al. 2022b). Although significant progress has been made in understanding the mechanisms driving liver metastasis in CRC, particularly the role of HSCs in promoting migration, angiogenesis, and modulating the immune microenvironment, the full underlying mechanism remains an active area of research. Accordingly, we postulated that cancer-derived exosomes might regulate the activation state of HSCs and consequently influence the metastatic potential and proliferative progression of colorectal cancer cells. To explore this theory, we established an indirect co-culture assay. In this experiment, HSCs were treated with exosomes from CRC cell lines that had either miR-1246 knocked down or overexpressed, followed by co-culturing with CRC cells (Fig. 3A). The subsequent transwell assay findings illustrated that LX2, upon activation by CRC-secreted exosomal miR-1246, had a pronounced metastasis-facilitating effect on CRC cells (Fig. 3B, C). Next, EDU assay results revealed that LX2 cells, after ingesting exosomes from the miR-1246 overexpression group, bolstered the proliferation of CRC cells. Conversely, the knockdown group displayed an opposing tendency (Fig. 3D, E). Analogously, the transfection of miR-1246 mimics into LX2 cells enhanced the migration and proliferation of CRC cell lines in subsequent tests (Supplementary Fig. 2A, B). Collectively, these results provide compelling evidence that CRC-secreted exosomal miR-1246 can activate HSCs, thereby promoted CRC migration and proliferation in vitro experiment.

### INSIG1 is a functional target of miR-1246 in HSCs

**Fig. 4:**
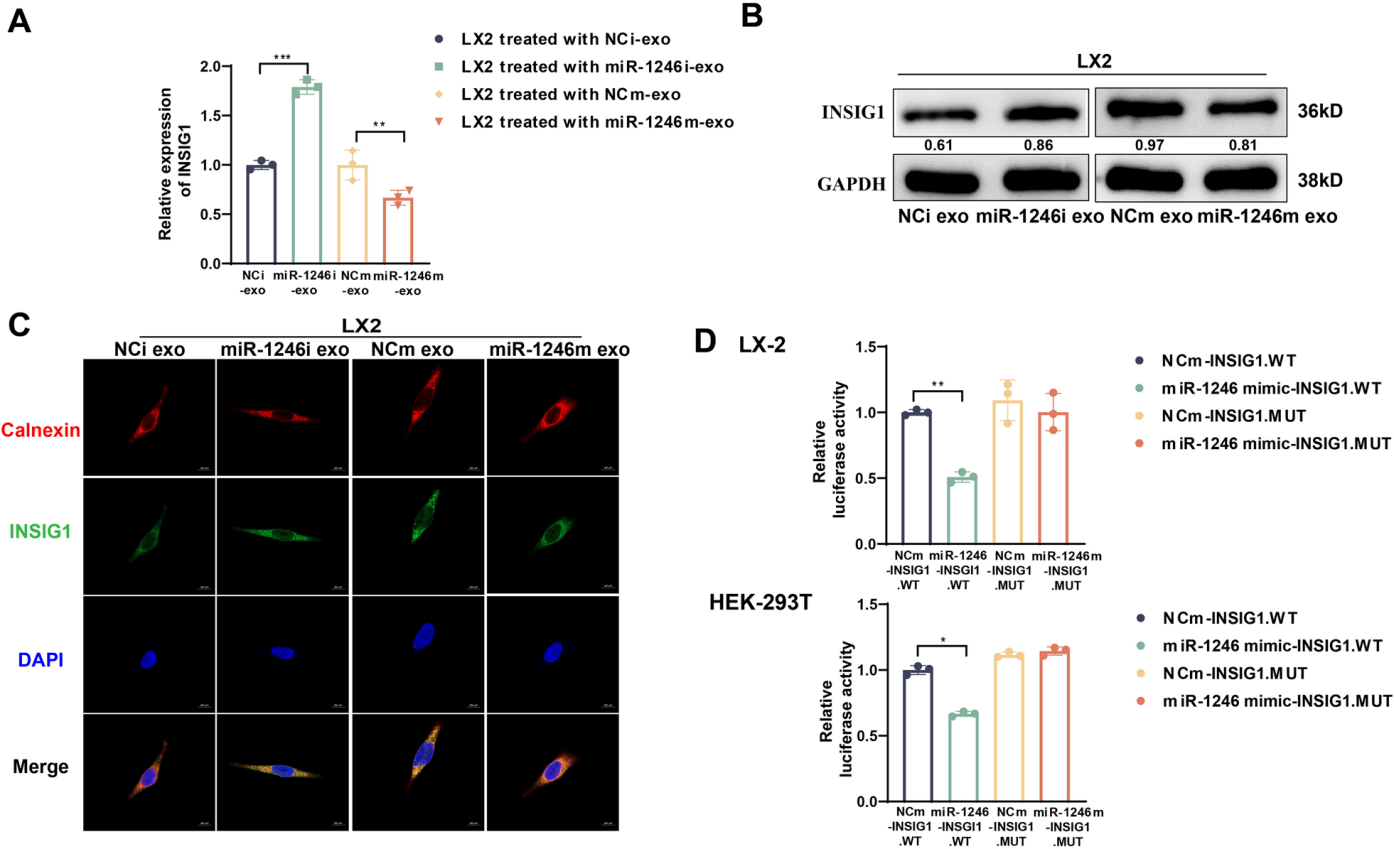
INSIG1 is a functional target of miR-1246 in HSCs. **A**, **B** Western blot and RT-PCR detection the reduced expression of INSIG1 in HSCs treated with CRC overexpressing miR-1246 exosomes. Mean ± SEM (n = 3). **C** Representative immunofluorescence images for in HSCs stimulated by CRC exosomes. Scale bars:20 µm. Mean ± SEM (n = 7). **D** Verification of luciferase activities in HEK293T and LX2 cells. Mean ± SEM (n = 3)

To explore how miR-1246 modulated the activation of HSCs. Based on the base-pairing principle, we employed three authoritative mRNA target prediction algorithms (miRDIP, miRmap, and Targetscan 8.0) to identify potential downstream targets of miR-1246. We found that INSIG1 was shared as a common target across all three databases (Supplementary Fig. 3A). Research reports have indicated that a decrease in INSIG1 disrupts its equilibrium with SREBP cleavage-activating protein (Scap), leading to an imbalance in the SREBP2-mediated cholesterol system in HSCs and heightening the sensitivity to TGF-β activation (Tomita et al. 2014). This accumulation of free cholesterol might trigger relevant pathways to activate HSCs (Musso et al. 2013; Lin et al. 2009). Consistent with prediction, both Western blot and RT-PCR results demonstrated a profound decrease in the expression of INSIG1 in LX2, following treatment with SW480-miR-1246m exosomes, while treatment with HCT116-miR-1246i exosomes resulted in elevated INSIG1 expression in LX2 (Fig. 4A, B). ICC assays further revealed a significantly reduced average fluorescence intensity in HSCs after receiving exosomes from the miR-1246 overexpressed CRC groups (Fig. 4C, Supplementary Fig. 3B). Co-localization of INSIG1 with Calnexin demonstrates that it is predominantly expressed on the cellular endoplasmic reticulum. To further substantiate these findings, we constructed an INSIG1 wild-type luciferase reporter plasmid (WT) containing the miR-1246 binding region 3utr (INSIG1-3UTR (mir1246)). Simultaneously, we deployed a plasmid with a mutated sequence of the miR-1246 binding site (MUT) to serve as a negative control 3utr (INSIG1-3UTR (mir1246)-mut) (Supplementary Fig. 3C). Notably, the luciferase activity of the 3′UTR of INSIG1 was suppressed by miR-1246 mimics in the INSIG1-WT group, with no observable changes in the INSIG1-MUT group in both HEK293T and LX2 cells (Fig. 4D). Collectively, our data strongly suggests that CRC-secreted exosomal miR-1246 specifically targets INSIG1 in HSCs.

### CRC-secreted exosomal miR-1246 promotes free cholesterol accumulation by targeting INSIG1 and regulates the TLR4/NF-κB/TGF-β pathway to activate HSCs

**Fig. 5:**
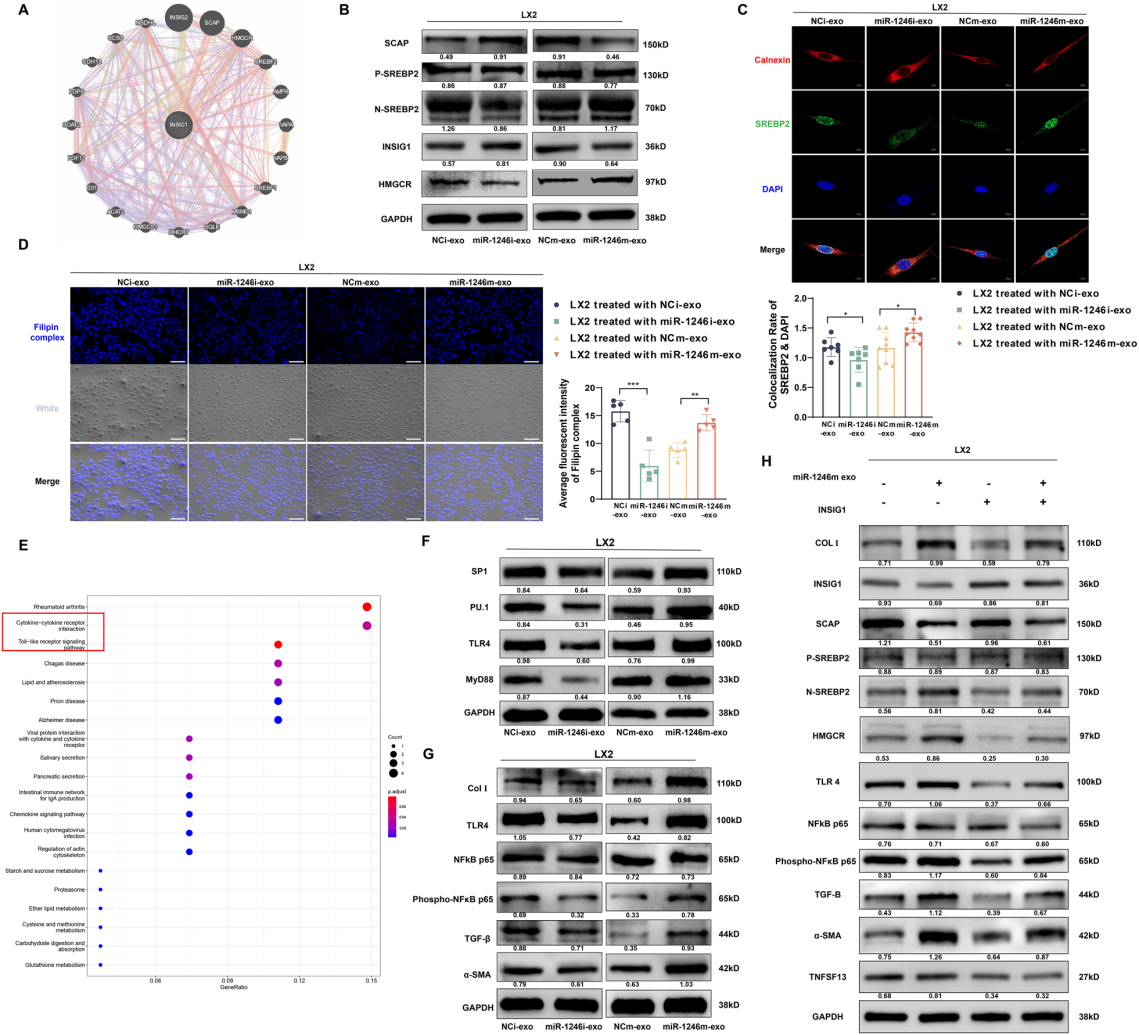
CRC-secreted exosomal miR-1246 promotes free cholesterol accumulation by targeting INSIG1 and regulates the TLR4/NF-κB/TGF-β pathway to activate HSCs. **A** The neuronal network of INSIG1-interacting proteins by using the GENEMANIA database. **B** Western blot validation of SREPB2 and SCAP expression in HSCs stimulated by CRC exosomes. **C** Representative immunofluorescence staining images and qualification of nuclear SREBP2. Scale bars: 20 µm. Mean ± SEM (n = 7). **D** Representative Philippine complex III staining images and qualification of free cholesterol level. Scale bars:100 µm. Mean ± SEM (n = 5). **E** Transcriptome sequencing results of HSCs stimulated with miR-1246 mimic. **F** Western blot confirms TLR4 complex expression in HSCs stimulated by CRC exosomes. **G** Western blot validation of TLR4/NF-κB/TGF-β pathway expression in HSCs stimulated by CRC exosomes. **H** Validation of the association between CRC-derived exosomal miR-1246 target INSIG1 and active TLR4/ NF-κB/ TGF-β pathway by Western Blot rescue experiment

To elucidate the mechanism through which miR-1246-INSIG1 aids in the activation of HSCs, we constructed a neuronal network of INSIG1-interacting proteins with the assistance of the GENEMANIA database. This revealed the principal role of INSIG1 as a regulating protein in cholesterol metabolism (Fig. 5A, Supplementary Fig. 4A). There are reports suggesting that INSIG1 is critical for retaining the SREBP cleavage-activating protein (SCAP) in the ER and stabilizing the SREBP-SCAP complex (Wang et al. 2022). As we hypothesized, HSCs primed with overexpressed miR-1246 exosomes exhibited reduced SCAP and increased nuclear SREBP2 expression (Fig. 5B). HMGCR was also detected to reflect the rate of cholesterol synthesis (Supplementary Fig. 4B). Concurrently, ICC assays displayed that exosomal miR-1246 notably elevated the localization of SREBP2 in the nucleus of LX2 (Fig. 5C). Filipin complex III staining, a fluorescent dye specifically binding to cholesterol, revealed an increase in free cholesterol levels in LX2 following treatment with SW480-miR-1246m exosomes, while treatment with HCT116-miR-1246i exosomes demonstrated adverse effects (Fig. 5D). To unravel potential mechanisms through which free cholesterol induces HSC activation, we executed mRNA-Seq to investigate differentially expressed genes in LX2 cell lines transfected with the miR-1246 mimic. We discovered that the Toll-like receptor signal pathway and Cytokines and Cytokine Receptor interaction were significantly enriched (Fig. 5E). Metabolic dysfunction-associated steatohepatitis (MASH), Free cholesterol accumulation amplifies Toll-like receptor 4 (TLR4) levels, thereby sensitive to TGF-β stimulation and leading to their activation (Trivedi et al. 2021). Interestingly, we observed that HSCs stimulated with miR-1246 overexpressed exosomes not only recorded an increase in TLR4 expression but also induced modification in Myd88, a classical TLR4 ligand. Moreover, the upregulation of TLR4 expression induced in this process was facilitated by modulation of its transcriptional regulators SP1 and PU.1 (Fig. 5F). This finding parallels a study by Xianjing Hu et al. where palmitic acid increases TLR4 expression in a PU.1-dependent manner, enhancing malignancy in CRC (Hu et al. 2021). TLR4 plays a pivotal role in the inflammatory response during liver fibrosis. It can activate NF-κB p65 to upregulate TGF-β expression, which is the most salient cytokine for the activation of HSCs (Ma et al. 2020; Wang et al. 2012). The Western blot illustrated that TLR4 pathway activation was associated with the phosphorylation of NF-κB p65 and an increase in the expression of TGF-β (Fig. 5G). RT-PCR showcased a similar trend (Supplementary Fig. 4C). Also, ICC assays showed that exosomal miR-1246 substantially increased the expression of TGF-β in LX2 (Supplementary Fig. 4D). The activation of HSCs by CRC-derived exosomal was mitigated by the overexpression of the INSIG1 plasmid (Fig. 5H). In summary, CRC-secreted exosomal miR-1246 suppressed INSIG1, prompting SREBP2 translocation into the nucleus, thereby facilitating free cholesterol synthesis. The accumulation of free cholesterol subsequently activated HSCs via the TLR4/ NF-κB /TGF-β pathway.

### aHSCs facilitate CRLM by activation of the TNFSF13/TNFRSF13B axis

**Fig. 6:**
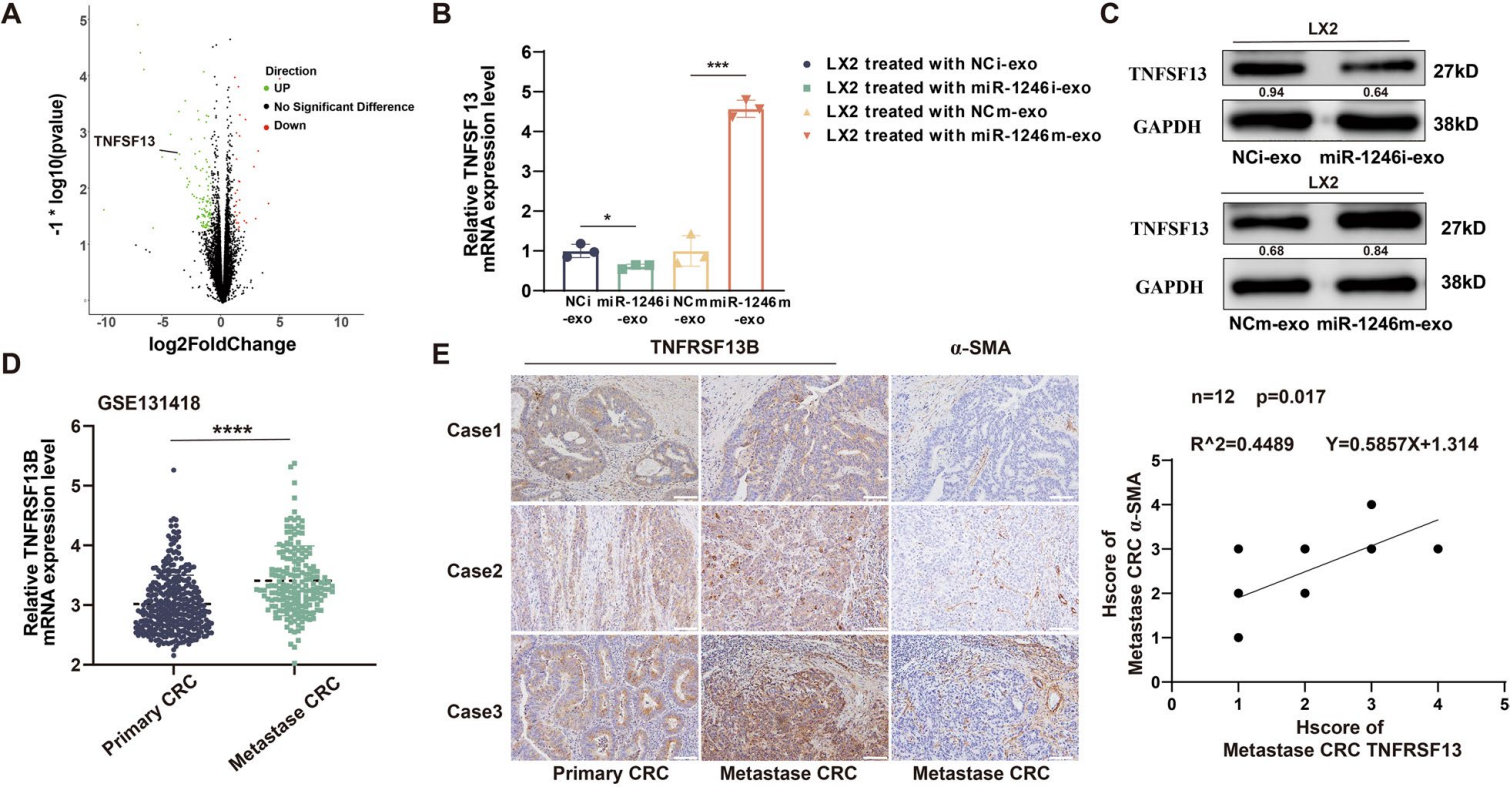
aHSCs facilitate CRLM by activation of the TNFSF13/ TNFRSF13B axis. **A** Display of TNFSF13 upregulation among differential genes in the volcano plot. **B**, **C** Verification of TNFSF13 expression in LX2 cells cultured with exosomes harvested from lentivirally transfected HCT116/SW480 cells, as shown by Western Blot and RT-PCR analyses. Mean ± SEM (n = 3). **D** Separate analyses of TACI (TNFRSF13B) mRNA array data. GEO (accession number: GSE131418). **E** Representative IHC images and quantitative analysis are shown. Scale bars: 50 µm. Mean ± SEM (n = 12)

Several cytokines have been pinpointed as being notably upregulated within aHSCs, contributing to the augmentation of the tumor microenvironment (Tsuchida and Friedman 2017; Higashi et al. 2017). We speculate that aHSCs may drive CRLM through secreting cytokines. Wang's research highlighted that TNFSF13 enhances the growth and metastatic potential of CRC, and higher expression suggests suboptimal prognosis and multi-organ metastasis in CRC patients (Nowacka and Jabłońska 2021; Wang et al. 2011). Corroborating our hypothesis, our RNA-Seq results indicated a significant enrichment of cytokines and receptor pathways (Fig. 5E). Moreover, the volcano plot demonstrated that TNFSF13 was the most significantly upregulated among all differentially expressed cytokines (Fig. 6A). Subsequently, LX2 cells were primed with miR-1246 overexpressed or knocked down exosomes from CRC cell lines, respectively. RT-PCR and Western blot outcomes revealed that miR-1246 overexpression or knockdown correspondingly enhanced or abolished TNFSF13 expression (Fig. 6B, C). Furthermore, receptors for TNFSF13 (Mackay et al. 2003; Baert et al. 2021), such as TACI (TNFRSF13B), BAFF, and BCMA, were separately analyzed in GSE131418. Notably, TNFRSF13B exhibited a higher expression in liver metastases compared with primary CRC instances (Fig. 6D, Supplementary Fig. 5A). These findings imply a more pronounced role of TNFRSF13B in the microenvironment of CRLM. We also observed that TNFRSF13B was positively correlated with the expression of α-SMA in patients with CRLM (Fig. 6E). As a corollary, we propose that TNFSF13 secreted by aHSCs cells could potentially drive CRC colonization and progress malformation via the TNFSF13/TNFRSF13B axis.

### CRC-secreted exosomal miR-1246 promotes CRC liver metastasis in vivo

**Fig. 7:**
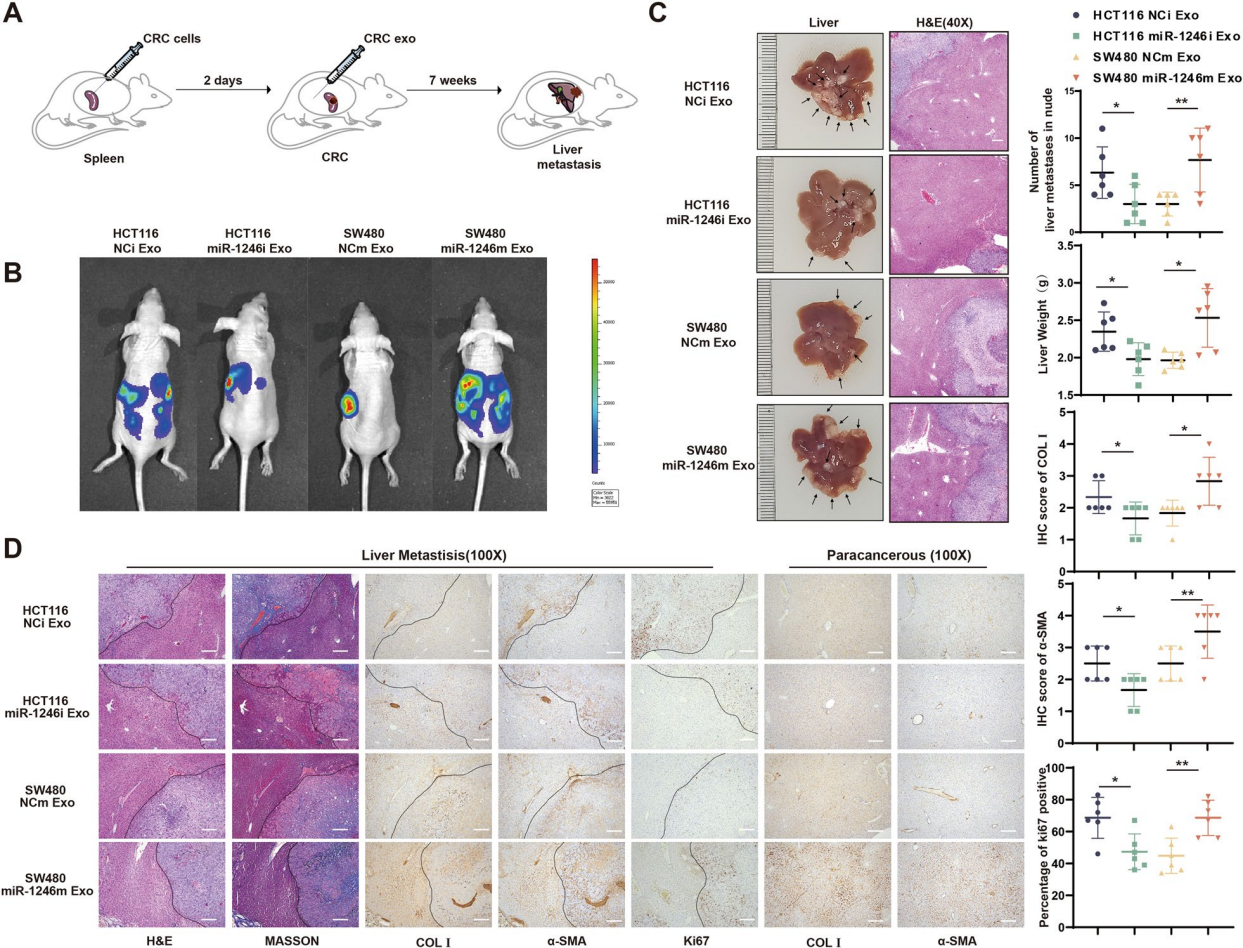
CRC-secreted exosomal miR-1246 promotes CRC liver metastasis in vivo. **A** The nude mouse models of CRC liver metastasis were established using HCT116 cells injected into the spleen's dorsal membrane and repeated intraperitoneal injections of lentivirus-stabilized exosome cell lines. **B** Qualification of bioluminescent imaging (BLI) and representative images of CRLM. (n = 6). **C** Quantification of liver metastases (indicated by arrows) and liver weight in nude mice. Mean ± SEM (n = 6). H&E staining are shown. Scale bars: 200 µm. **D** Representative illustrations of H&E, Masson and IHC staining. Scale bars: 100 µm Mean ± SEM (n = 6)

The intrasplenic injection model presents a reliable and controlled method for inducing liver metastasis in mice, offering a gateway to study the microenvironment of liver metastases. We employed this model to further investigate how exosomal miR-1246 might regulate CRC liver metastasis in vivo by injecting HCT116 cells intrasplenically and then intraperitoneally injecting exosomes from CRC cell lines overexpressing or knocking down miR-1246 on a weekly basis (Fig. 7A). We then monitored liver metastases using in vivo bioluminescence imaging and H&E staining (Fig. 7B, C). Our observations disclosed a significant increase in CRC liver metastases in mice receiving overexpressed miR-1246 exosomes. Additionally, a more robust liver appearance and weight in this group suggested enhanced CRC colonization and growth compared to the NCm group. In contrast, the results for the group receiving the knockdown miR-1246 exosomes were reversed. H&E and Masson staining revealed that mice receiving miR-1246 overexpressing exosomes exhibited higher metastasis rates and greater collagen fiber accumulation (Fig. 7D). Immunohistochemistry (IHC) results showed that the group receiving exosomes from the miR-1246 overexpression displayed more pronounced levels of α-SMA and ColI expression, indicative of stromal remodeling in the paracancerous normal liver tissue. Furthermore, the expression of liver α-SMA in the mice was found to directly correlate with the number and proliferation of liver metastatic tumor cells. Taken together, our data corroborate that CRC-secreted exosomal miR-1246 triggers HSCs activation, thereby promoting CRC metastasis.

## Discussion

CRLM remains the leading cause of cancer-related mortality, driven by the complex interactions between tumor cells and the liver microenvironment. This presents a significant challenge in current cancer treatment strategies. An increasing body of evidence suggests that exosomes play a crucial role in facilitating communication between cancer cells and their microenvironment (Zhang et al. 2020). In our research, we discovered that CRC-secreted exosomes were specifically targeted for hepatic uptake, indicating a more intricate interaction between colorectal cancer and the hepatic microenvironment. Exosomes are typically vesicle-like structure with a diameter of approximately 100 nm, encapsulating various biological materials such as nucleic acids and proteins (Zhang et al. 2015). These structures have been reported to play key roles in intercellular communication, maintaining organ homeostasis, and contributing to disease development (Kalluri and Lebleu 2020). Numerous studies suggest that cancer cell-derived exosomes contribute to CRLM by modulating the metastatic microenvironment (Mannavola et al. 2020; Dai et al. 2020). However, the molecular mechanism by which specific exosomes CRLM remain poorly understood.

In our study, we present evidence that CRC-secreted miR-1246 can be delivered to hepatic stellate cells (HSCs) via exosomes. Upon receiving these cancer-derived exosomes, HSCs are activated, leading to the formation of a tumor-promoting microenvironment that facilitates CRLM progression. We present evidence that CRC-secreted miR-1246 can be delivered to HSCs via exosomes. Upon receiving these cancer-derived exosomes, HSCs are activated, leading to the formation of a tumor-promoting microenvironment that facilitates CRLM progression.

MicroRNAs represent a class of small non-coding RNAs that regulate gene expression post-transcriptionally by targeting mRNAs, contributing to intercellular communication (Hussen et al. 2021). For example, exosomal miR-205, secreted by ovarian cancer cells, targets PTEN expression in HUVEC cells, thereby promoting angiogenesis and metastasis (He et al. 2019). Similarly, gastric cancer-derived exosomal miR-519a-3p targets DUSP2, activating the MAPK/ERK pathway and inducing macrophage M2-like polarization, which accelerates gastric cancer liver metastasis (Qiu et al. 2022). These findings demonstrate that cancer cell-derived exosomes are absorbed by endothelial cells and macrophages, leading to molecular phenotypic changes that exacerbate cancer progression.

In a similar context, miR-1246, a pro-oncogenic miRNA, has been linked to poor prognosis in CRC patients and may serve as a non-invasive biomarker for early CRC diagnosis (Desmond et al. 2019; Pruseth et al. 2021).

Consistent with our research, immunohistochemistry (IHC) results showed high expression of miR-1246 in CRC patients, aligning with both cell line exosomal and endogenous miR-1246. A pan-cancer miRNA matrix analysis revealed that miR-1246 is more highly expressed in colorectal cancer compared to other cancer types, suggesting superior specificity and sensitivity for predicting CRC development. Furthermore, we observed that PKH67-labeled CRC exosomes were predominantly localized in the liver, and fluorescently labeled exosomes were visibly taken up by HSCs. These observations suggest that CRC-generated miR-1246 may be transferred to the liver and assimilated by HSCs in an organ-specific manner, thereby contributing to the formation of a pro-metastatic microenvironment.

Stellate cells are highly adaptable and play critical roles in liver growth, immune responses, inflammation, energy balance, and metabolic homeostasis (Trivedi et al. 2021). HSCs are particularly important in establishing the metastatic microenvironment by recruiting circulating CRC tumor cells and promoting liver metastases (Yang et al. 2021). Moreover, chemokines and cytokines released by HSCs attract inflammatory and immune cells, together shaping the liver's immune microenvironment (Tsilimigras et al. 2021). A cascade of pro-tumorigenic factors, including IL6, VEGFA, IL11, PDGFA, and CXCL12, further amplify the formation of complex microenvironments that support tumor cell colonization and proliferation, exacerbating the progression of CRLM (Dou et al. 2018; Coulouarn 2015). Understanding of the mechanisms by which activated HSCs initiate tumor metastasis in the liver could offer valuable insights for CRLM treatment.

In our study, we demonstrated that CRC-derived exosomal miR-1246 activates HSCs, and that silencing the target gene INSIG1 leads to metabolic reprogramming within the tumor microenvironment (TME), contributing to the establishment of a metastatic landscape in CRLM. Existing evidence demonstrates that exosomes transport bio-functional molecules to recipient cells, reprogramming their metabolism to promote cancer progression, angiogenesis, metastasis, drug resistance, and immunosuppression (Yang et al. 2020). For instance, Raudenska et al. reported that CRC regulates cholesterol distribution within cancer-associated fibroblasts, influencing tumor progression (Raudenska et al. 2020). Similarly, enhanced cholesterol and steroid biosynthesis in cancer-associated fibroblasts accelerates prostate tumor growth and development (Neuwirt et al. 2020). These alterations in cholesterol metabolism serve as a catalyst for malignant progression in cancer cells. In our investigation, we found that CRC-derived exosome miR-1246 silences the INSIG1 gene in recipient HSCs, leading to the accumulation of free cholesterol. Consistent with Teratani et al.’s research, this accumulation triggers HSC activation through the TLR4 pathway (Tomita et al. 2014). While LDLR and miR-33a are recognized for their role in enhancing HSC sensitivity to TGF-β by preventing TLR4 degradation through the endosomal-lysosomal system, our results highlight the activation of the TLR4 ligand pathway. Furthermore, the upregulation of TLR4 expression could be linked to the influence of free cholesterol on its transcriptional overseer, PU.1. This aligns with Xianjing Hu and colleagues' study wherein palmitic acid modulates TLR4 expression in a PU.1-dependent manner, adjusting cancer metabolism in colorectal cancer under high-fat dietary conditions (Hu et al. 2021). Subsequent to these observations, we identified that aHSCs release TNFSF13, facilitating the recruitment of circulating CRC and furthering the malignant progression of liver metastatic CRC tumors. These results underscore the crucial role of CRC-derived exosomes in mediating the phenotypic transformation and interplay of HSCs within tumor cells and the liver metastasis microenvironment. Importantly, these findings could enhance our ability to predict CRLM risk and potentially inform the development of innovative clinical intervention strategies.

In conclusion, our study demonstrated that CRC-derived exosomal miR-1246 silences INSIG1 expression, promoting SREBP2 nucleation and increasing free cholesterol synthesis. This accumulation of free cholesterol activates the TLR4/NF-κB/TGF-β pathway, leading to the activation of hepatic stellate cells (HSCs). Subsequently, activated HSCs (α-HSCs) secrete TNFSF13, which recruits and promotes tumor progression through the TNFSF13/TNFRSF13B axis. These findings reveal novel molecular mechanisms underlying the interactions between CRC cells and HSCs, shedding light on the role of cholesterol metabolic reprogramming and ligand-receptor pathways in the development of colorectal cancer liver metastases. Our study provides new predictive metrics and potential targets for clinical intervention in CRLM.

## Supplementary Information

Supplementary Material 1: Fig. 1. Up-regulation of exosomal miR-1246 is related to liver metastatic progression and HSCs activation in CRLM patients.

Supplementary Material 2: Fig. 2. HSCs activated by miR-1246 promote CRC proliferation and migration.

Supplementary Material 3: Fig. 3. INSIG is a functional target of miR-1246 in HSCs.

Supplementary Material 4: Fig. 4. CRC-secreted exosomal miR-1246 regulates the TLR4/TGF-β pathway to activate HSCs.

Supplementary Material 5: Fig. 5. Expression of TNFSF13 classical ligand BCMA and BAFF-R during CRLM.

Supplementary Table 1. Lentivirus and mimic sequences.

Supplementary Table 2. Primer sequences for RT-PCR.

## Data Availability

No datasets were generated or analysed during the current study.
