# Supplementary figures and images for: Cancer-secreted exosomal miR-1246 promotes colorectal cancer liver metastasis by activating hepatic stellate cells
Source: Mol Med. 2025 Feb 20;31:68. doi: 10.1186/s10020-025-01112-w (PMC11841005; doi:10.1186/s10020-025-01112-w)

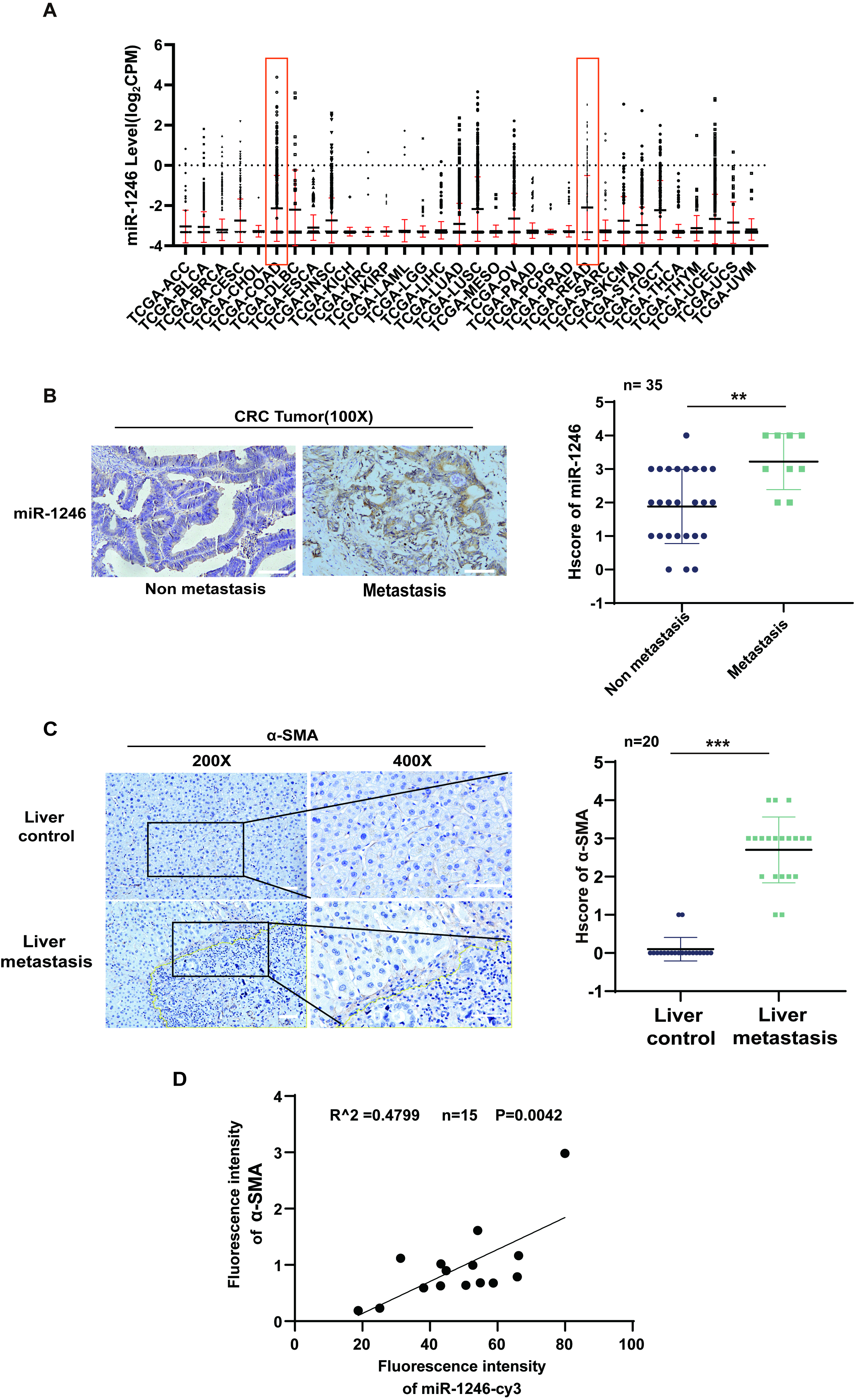

Supplement: Supplementary file 1 — Supplementary Material 1: Fig. 1. Up-regulation of exosomal miR-1246 is related to liver metastatic progression and HSCs activation in CRLM patients. [file 10020_2025_1112_MOESM1_ESM.tif]

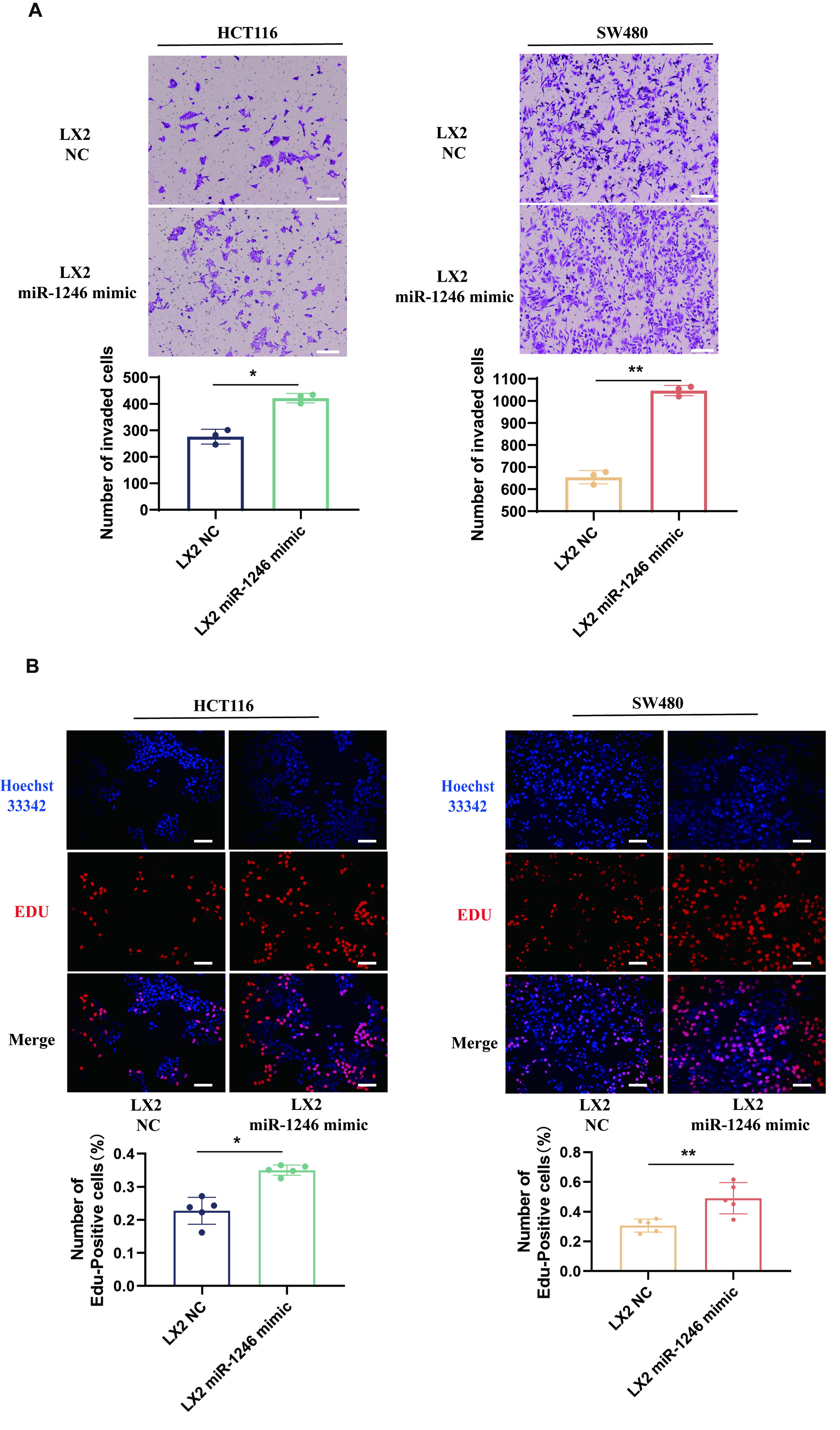

Supplement: Supplementary file 2 — Supplementary Material 2: Fig. 2. HSCs activated by miR-1246 promote CRC proliferation and migration. [file 10020_2025_1112_MOESM2_ESM.tif]

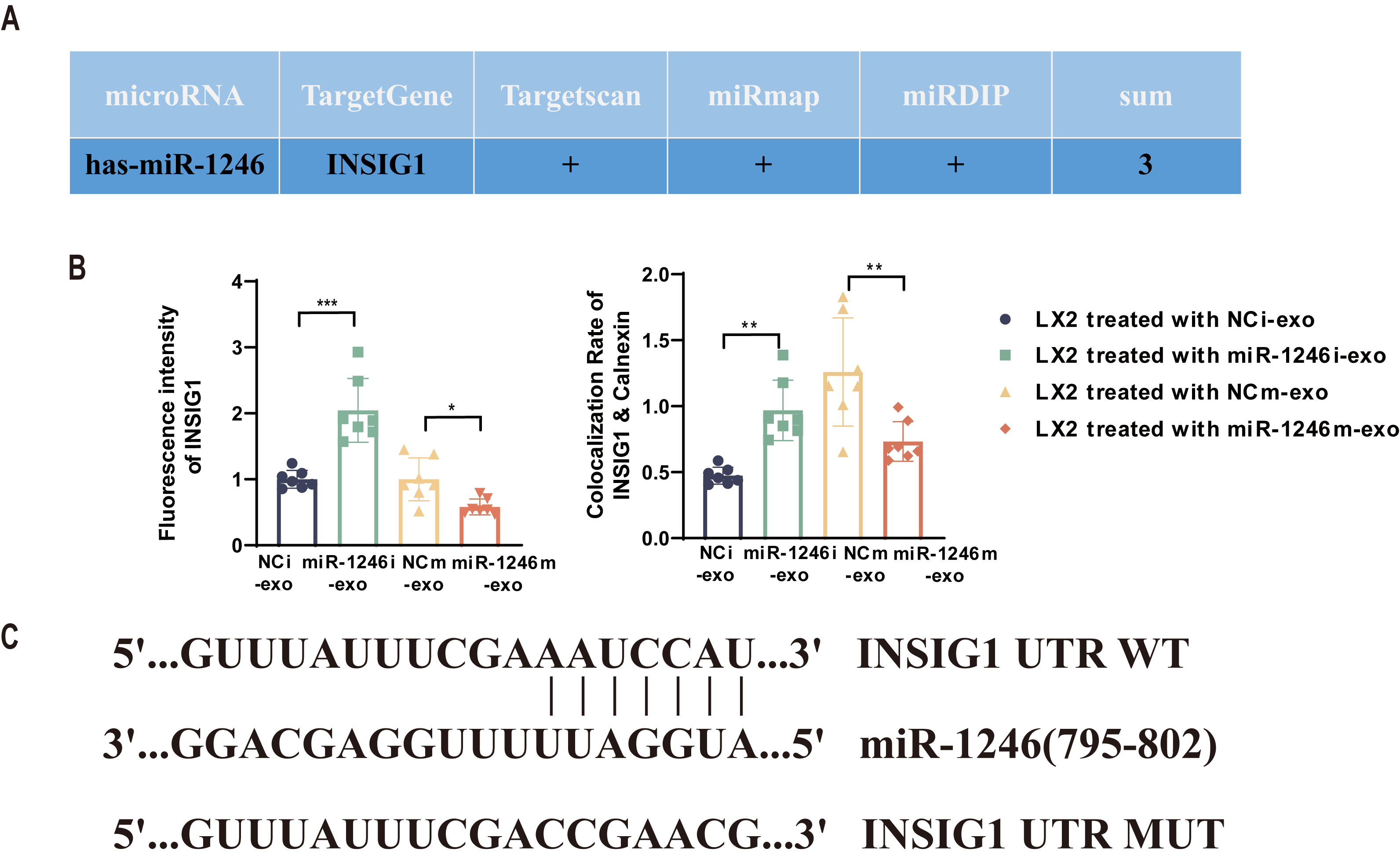

Supplement: Supplementary file 3 — Supplementary Material 3: Fig. 3. INSIG is a functional target of miR-1246 in HSCs. [file 10020_2025_1112_MOESM3_ESM.tif]

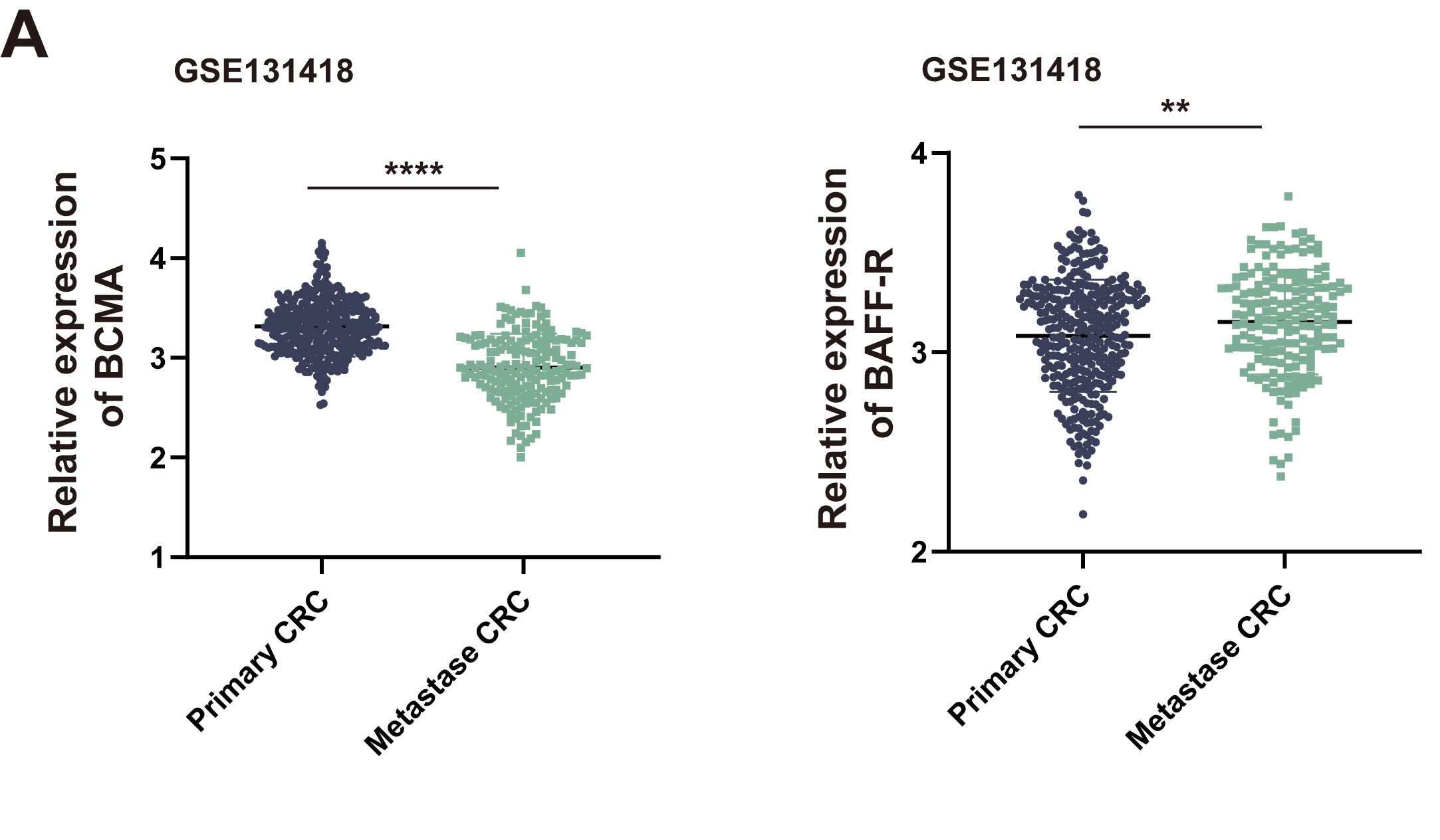

Supplement: Supplementary file 5 — Supplementary Material 5: Fig. 5. Expression of TNFSF13 classical ligand BCMA and BAFF-R during CRLM. [file 10020_2025_1112_MOESM5_ESM.tif]
