# Supplementary material for: Cancer-secreted exosomal miR-1246 promotes colorectal cancer liver metastasis by activating hepatic stellate cells
Source: Mol Med. 2025 Feb 20;31:68. doi: 10.1186/s10020-025-01112-w (PMC11841005; doi:10.1186/s10020-025-01112-w)
Supplement: Supplementary file 6 — Supplementary Table 1. Lentivirus and mimic sequences. [file 10020_2025_1112_MOESM6_ESM.docx]

Supplementary Table 1. Lentivirus and mimic sequences.

| Name | Sequence(5’→3’) |
| --- | --- |
| miR-1246 inhibitor lentivirus |  |
| miR-1246-inhibition-a | AATGGATTTTTGGAGCAGG |
| miR-1246-inhibition-b | CCTGCTCCAAAAATCCATT |
| miR-1246 mimics lentivirus | UGUAUCCUUGAAUGGAUUUUU  GGAGCAGGAGUGGACACCUGA  CCCAAAGGAAAUCAAUCCAUAG  GCUAGCAAU |
| miR-1246 mimics sense  miR-1246 mimics antisense | AAUGGAUUUUUGGAGCAGG  CCUGCUCCAAAAAUCCAUU |
