# Supplementary material for: Cancer-secreted exosomal miR-1246 promotes colorectal cancer liver metastasis by activating hepatic stellate cells
Source: Mol Med. 2025 Feb 20;31:68. doi: 10.1186/s10020-025-01112-w (PMC11841005; doi:10.1186/s10020-025-01112-w)
Supplement: Supplementary file 7 — Supplementary Table 2. Primer sequences for RT-PCR. [file 10020_2025_1112_MOESM7_ESM.docx]

Supplementary Table 2. Primer sequences for RT-PCR.

| Primer | Sequence (5’→3’) |
| --- | --- |
| hsa-miR-1246 | AATGGATTTTTGGAGCAGGAA |
| U6-F | GGAACGATACAGAGAAGATTAGC |
| U6-R | TGGAACGCTTCACGAATTTGCG |
| GAPDH-F | CCATCAATGACCCCTTCATTGACC |
| GAPDH-R | GAAGGCCATGCCAGTGAGCTTCC |
| INSIG1-F | CCTGGCATCATCGCCTGTT |
| INSIG1-R | AGAGTGACATTCCTCTGGATCTG |
| Sp1-F  Sp1-R | GGCTACCCCTACCTCAAAGG  CACAACATA CTGCCCACCAG |
| PU.1-F | GTGCCCTATGACACGGATCTA |
| PU.1-R  Myd88-F  Myd88-R  TIRAP-F  TIRAP-R  TLR4-F  TLR4-R  TGF-β-F  TGF-β-R  HMGCR-F  HMGCR-F  TNFSF13-F  TNFSF13-R | AGTCCCAGTAATGGTCGCTAT  GGCTGCTCTCAACATGCGA  CTGTGTCCGCACGTTCAAGA  GACCCCTGGTGCAAGTACC  CGACGTAGTACATGAATCGGAG  AGACCTGTCCCTGAACCCTAT  CGATGGACTTCTAAACCAGCCA  GGCCAGATCCTGTCCAAGC  GTGGGTTTCCACCATTAGCAC  TGATTGACCTTTCCAGAGCAAG  CTAAAATTGCCATTCCACGAGC  GCTGCCCCATGTTCAGCCTC  CAGGCCCCACACACACTTCC |
